# Building on facilitators and overcoming barriers to implement active tuberculosis case-finding in Nepal, experiences of community health workers and people with tuberculosis

**DOI:** 10.1186/s12913-021-06290-x

**Published:** 2021-04-01

**Authors:** Olivia Biermann, Kritika Dixit, Bhola Rai, Maxine Caws, Knut Lönnroth, Kerri Viney

**Affiliations:** 1 Department of Global Public Health, WHO Collaborating Centre on Tuberculosis and Social Medicine, Karolinska Institutet, Tomtebodavägen 18a, 17177 Stockholm, Sweden; 2 Birat Nepal Medical Trust, Lazimpat, Kathmandu, Nepal; 3 Department of Clinical Sciences, Liverpool School of Tropical Medicine, Pembroke Pl, Liverpool, L3 5QA UK; 4 Research School of Population Health, College of Health and Medicine, Australian National University, Canberra, Australia

**Keywords:** Tuberculosis, Active case-finding, Implementation, Nepal, Facilitators, Barriers

## Abstract

**Background:**

Nepal has a high burden of undetected tuberculosis (TB). In line with the World Health Organization’s End TB Strategy, the National TB Programme promotes active case-finding (ACF) as one strategy to find people with TB who are unreached by existing health services. The IMPACT TB (Implementing proven community-based active TB case-finding intervention) project was implemented in four districts in Nepal, generating a substantial yield of previously undetected TB. We aimed to identify the facilitators and barriers linked to the implementation of ACF within IMPACT TB, as well as how those facilitators and barriers have been or could be addressed.

**Methods:**

This was an exploratory qualitative study based on 17 semi-structured key-informant interviews with people with TB who were identified through ACF, and community health workers who had implemented ACF. Thematic analysis was applied in NVivo 11, using an implementation science framework developed by Grol and Wensing to classify the data.

**Results:**

We generated five main themes from the data: (1) ACF addressed the social determinants of TB by providing timely access to free healthcare, (2) knowledge and awareness about TB among people with TB, communities and community health workers were the ‘oil’ in the ACF ‘machine’, (3) trust in community health workers was fundamental for implementing ACF, (4) community engagement and support had a powerful influence on ACF implementation and (5) improved working conditions and enhanced collaboration with key stakeholders could further facilitate ACF. These themes covered a variety of facilitators and barriers, which we divided into 22 categories cutting across five framework levels: innovation, individual professional, patient, social context and organizational context.

**Conclusions:**

This study provides new insights into facilitators and barriers for the implementation of ACF in Nepal and emphasizes the importance of addressing the social determinants of TB. The main themes reflect key ingredients which are required for successful ACF implementation, while the absence of these factors may convert them from facilitators into barriers for ACF. As this study outlined “how-to” strategies for ACF implementation, the findings can furthermore inform the planning and implementation of ACF in Nepal and similar contexts in low- and middle-income countries.

**Supplementary Information:**

The online version contains supplementary material available at 10.1186/s12913-021-06290-x.

## Introduction

An estimated 10 million people fell ill with tuberculosis (TB) in 2019, of whom 2.9 million people were not diagnosed or reported to the World Health Organization (WHO) [1]. The WHO End TB Strategy emphasizes active case-finding (ACF) as one approach to finding the people with TB who are currently being missed by health services [2]. WHO defines ACF as the systematic identification of people with presumed active TB, in a predetermined target group, using tests, examinations or other procedures that can be applied rapidly [3]. ACF is synonymous with systematic screening for active TB, while it denotes screening outside of health facilities [3]. Compared to “passive case-finding” (PCF), ACF has been shown to find more people with TB at an earlier stage of disease [4, 5]. ACF ideally supplements PCF; the latter being the standard approach to TB case-finding, relying on people seeking care when they have signs and symptoms of TB [6]. The potential benefits and harms of ACF must be assessed in any given context [3, 7].

Nepal is a lower middle-income country in South Asia. The 2018 national TB prevalence survey showed an annual incidence of 245 TB cases per 100,000 population, which was almost 1.6 times higher than previously estimated [8]. Notably, more than 70% of TB cases found during the TB prevalence survey reported no symptoms of TB [8]. As people without symptoms are unlikely to seek healthcare, this large number of asymptomatic cases is expected to contribute to the gap. According to recent estimates, 30–50% of incident TB cases are not notified [9]. Other factors that have been shown to delay TB diagnosis in Nepal include patient income and occupation [10], as well as long distances to health centers, poor road conditions and costs associated with travelling, lack of awareness about TB, and shortage of personnel and equipment [11].

The Nepal National TB Programme aims to increase TB case notifications by improving diagnosis and by screening household contacts, children under the age of 5 years and other vulnerable groups, such as people living with human immunodeficiency virus (HIV) and diabetes mellitus [9]. ACF projects in Nepal date back to 1982 [12]. In the past decade, different ACF models have been implemented in the country, e.g. one ACF project among people living with HIV showed that a peer-led approach to ACF resulted in a high participation rate and the identification of people with TB [13]. Eight ACF projects in Nepal have been funded by TB REACH, a case-finding initiative sponsored largely by Global Affairs Canada and coordinated by the Stop TB Partnership [14]. For example, one TB REACH project in Nepal focused on people living with HIV, household contacts and urban slum dwellers, resulting in a substantial yield of TB cases [15].

ACF requires appropriate coordination and integration within a given health system [3, 16], while it is often constrained by limited human and financial resources [17, 18]. However, many other factors influence ACF implementation [19]. For example, the experience, skills and motivation of those implementing ACF have been illustrated as strong influencing factors for ACF implementation [20–22]. The evidence base remains limited when it comes to understanding not only what the facilitators and barriers are, but also how to address them in a given context [19].

IMPACT TB (Implementing proven community-based active TB case finding intervention; www.impacttbproject.org) was a Horizon 2020-funded project on ACF in Vietnam and Nepal (2017–2019). In Nepal, ACF was implemented through community health workers (including community volunteers, Female Community Health Volunteers and Community Mobilizers). The ACF model applied during IMPACT TB generated a substantial yield of TB cases (*n* = 1193), TB notifications increased by 13% [23] and TB-related patient costs were significantly reduced [24]. Still, an important knowledge gap remains in terms of understanding the facilitators and barriers for implementing ACF as well as “how-to” strategies of ACF, which is the focus of this study.

## Methods

### Aim and design

This was an exploratory qualitative study based on semi-structured key-informant interviews [25]. We used the COREQ (COnsolidated criteria for REporting Qualitative research) Checklist [26] to report the study (Additional file 1).

The study aimed to identify the facilitators and barriers linked to the implementation of ACF within IMPACT TB, as well as how those facilitators and barriers have been or could be addressed.

### Setting of the study

The Birat Nepal Medical Trust (BNMT), a national non-government organization, implemented the IMPACT TB ACF model in four districts of Nepal with a high prevalence of poverty and TB: i.e. Dhanusha and Mahottari (in Province 2) and Chitwan and Makwanpur (in Province 3) [9]. Makwanpur is a hilly district with a limited road network, while the other districts are on the lowland plains (the Terai). The districts are challenged by pockets of high urban population density, poor health indicators and high rates of illiteracy [27].

The ACF model was implemented by Female Community Health Volunteers (from the government-appointed network) supplemented by additional community volunteers appointed by BNMT, and Community Mobilizers (also appointed by BNMT). Staff appointed by BNMT were recruited from the local community by IMPACT TB District Program Coordinators in close collaboration with the local health office (from here on jointly referred to as “community health workers”). In urban, semi-urban and hard-to-reach areas, community health workers screened household contacts of people with TB using a simple standardised oral symptom screening questionnaire (8 questions), transported sputum samples from anyone who was positive for one or more symptoms to local laboratories, supported people who were diagnosed with TB to enrol on TB treatment and conducted follow-up visits. Community health workers received incentives for visiting a TB patient and listing his or her contacts (Rs 225, approx. USD 1.90), for each sputum sample collected and transported to the local laboratory (Rs 180, approx. USD 1.50) and for each person identified with TB (Rs 200, approx. USD 1.70). Community Mobilizers supervised and monitored the work of the community volunteers and Female Community Health Volunteers and received an additional stipend to cover transportation costs. The overall coordination of stakeholders in the district, oversight of ACF implementation, planning and reporting was managed by District Program Coordinators. The Community Mobilizers and District Program Coordinators received salaries of Rs 10,000 (approx. USD 86) and Rs 63,000 (approx. USD 536) respectively. In *Makwanpur* and *Mahottari,* sputum smear microscopy was used for TB diagnosis, while the Xpert^®^ MTB/RIF assay was used in *Dhanusha* and *Chitwan.*

### Characteristics of the participants, recruitment and sample selection

Interviewees were purposively sampled and included two different stakeholder groups: (1) people diagnosed with TB, identified through ACF, and (2) community health workers implementing ACF. The people with TB had to be on TB treatment or have recently completed it to be approached for an interview. The community health workers were chosen amongst those with the longest working experience within IMPACT TB. Moreover, the community health workers had to be available during the days of the interviews while many were travelling as part of the sputum sample collection. In collaboration with the IMPACT TB District Program Coordinators and Community Mobilizers in the districts, KD and BR contacted potential interviewees, informed them about the study and set up interview appointments for those who agreed to be interviewed. All invited participants agreed to be interviewed, amounting to 17 interviews (Table 1). The interviews took an average of 31 minutes to complete.

**Table 1:** Overview and characteristics of the interviewees

Stakeholder groups	n	Female (% of study participants)	Male (% of study participants)
**People with TB identified through ACF**	**8**	**2 (25%)**	**6 (75%)**
**Community health workers implementing ACF**	**9**	**4 (44%)**	**5 (56%)**
Community volunteers	4	0 (0%)	4 (100%)
Female Community Health Volunteers	3	3 (100%)	0 (0%)
Community mobilizers	2	1 (50%)	1 (50%)
**Total**	**17**	**6 (35%)**	**11 (65%)**

### Data collection

KD and BR collected the data in June 2019 through semi-structured interviews. OB developed interview guides with open-ended questions for the community health workers and people with TB (Additional file 2) which KD, BR, MC, KL and KV provided feedback on. The interview guides covered questions on what interviewees considered to be both the positive and negative aspects of implementing/participating in ACF, and which aspects they thought could be improved, including how to improve them, in future ACF implementation efforts. The interview guides were translated into Nepali and were back translated into English. Being bilingual in English and Nepali, KD and BR ensured the quality of the translations. The first two interviews were conducted as pilot interviews after which the guides were revised in terms of the order and clarity of the questions. The pilot interviews were included in the analysis.

At the beginning of each interview, the participants were given/read background information about the study, provided written informed consent and received an incentive of Rs 500 (approx. USD 4.28) to compensate them for their time. The interviews were conducted in hotels, health facilities, homes of people with TB and the BNMT regional office in Hetauda, depending on the preference of the participant.

During nine of the interviews, KD or BR, OB and the respective interviewee were present. Four interviews were conducted in the local language Maithili; during those interviews a District Program Coordinator acted as interpreter from Maithili to Nepali. During one interview, family members were present. To avoid potential cultural or gender-related barriers in communication, KD (female) interviewed women with TB, while BR (male) interviewed men with TB.

No repeat interviews were carried out and no formal field notes were taken. The audio-recorded interviews were transcribed verbatim and translated into English by an independent public health professional fluent in both languages. We ensured the anonymity and confidentiality of the participants by coding the participants as numbers and removing all identifiers. Interviews were conducted until reaching information power [38].

To ensure the quality of data collection, OB, KD and BR travelled to the four IMPACT TB districts together during the period of data collection and held debriefings after each interview. Moreover, they presented the preliminary findings to the BNMT staff in July 2019, which provided an opportunity for critically reflecting on and validating the findings; the “how-to” aspects of ACF were emphasized as particularly valuable, which led to greater emphasis on those aspects in the subsequent presentation of the results (Table 3).

### Data analysis

We conducted a data-driven thematic analysis [28] in *NVivo 11*, employing a realist approach considering the whole data set and reporting experiences, meanings and the reality of interviewees. Choosing a realist approach meant that we focused on the manifest rather than the latent content of the interviews. OB, KD and BR jointly analysed the data. First, OB coded six interviews. She discussed and revised the codes with KD and BR, and then regularly revised them throughout the analysis. Second, a list of codes was developed that reflected the facilitators and barriers for ACF implementation. Codes were checked across the data set and new codes were added when identified. Third, the authors used an implementation science framework by Grol and Wensing [29] entitled “Barriers to and incentives for change at different levels of healthcare” to review and structure the data. The framework outlines how barriers and facilitators can be identified, categorized and used for the development of a tailored intervention strategy. It categorizes facilitators and barriers into levels: the innovation (in our case ACF), the individual professional, the patient, the social context, the organizational context, and the external environment (e.g. political factors). We did not describe the external environment in our analysis to keep the scope of the article strongly focused on local implementation rather than policy and political issues. Fourth, the categories were created from observed patterns of meaning in the data and the theoretical understanding gained during the previous step. Fifth, the findings were mapped onto the framework to show their contributions to it, and to compare and contrast perceptions across the different groups of interviewees. Finally, five themes were identified that captured the meaning and association between the categories. Table 2 provides an example of the coding process.

**Table 2:** Example of the coding process

Interviewee	Quote	Code	Category	Framework level	Theme
Interviewee #11, person with TB, Dhanusha	If (the volunteer) would not have come, then I would have kept roaming, would take medicines and tablets, would be cured or not.	Benefit for patient	Access to healthcare through ACF	Innovation	ACF addressed social determinants of TB by providing timely access to free healthcare.

## Results

We identified five main themes from the data: (1) ACF addressed social determinants of TB by providing timely access to free healthcare, (2) knowledge and awareness about TB among people with TB, communities and community health workers were the ‘oil’ in the ACF ‘machine’, (3) trust in community health workers was fundamental for implementing ACF, (4) community engagement and support had a powerful influence on ACF implementation and (5) improved working conditions and better collaboration with key stakeholders could further facilitate ACF. These themes covered a variety of facilitators and barriers, which we divided into 22 categories and structured based on Grol and Wensing’s five framework levels: innovation, individual professional, patient, social context and organizational context (Table 3). In the following section, we provide a narrative that summarizes the categories per framework level and provides examples of how to build on facilitators and overcome barriers.

**Table 3:** Categories divided by framework level and specified as facilitator/barrier, and “how-to” examples

Level		Categories	Facilitator	Barrier	Examples of how to build on facilitators and overcome barriers
**Innovation**	1	Access to healthcare through ACF	x		IMPACT TB increased access to healthcare through ACF
	2	Early detection of TB	x		IMPACT TB facilitated early detection through ACF
	3	Economic benefits	x		Economic benefits (e.g. through early diagnosis or by saving travel costs) helped increase participation in ACF
	4	Free testing and treatment	x		Free testing and treatment motivated participation in ACF
	5	Support for people with TB	x		Community health workers provided support, e.g. by being caring, providing counselling and conducting follow-ups
**Individual professional**	1	Dedication and motivation	x	x	Community health workers encouraged ACF participation, showed persistence and strong willingness to help others
	2	Experience and skills	x	x	Community health workers used communication, persuasion and interpersonal skills; community health workers were familiar with the local context
	3	Having a network that facilitates ACF	x		Community health workers used their networks, reputation and relationships (e.g. from working as teachers or social workers) to find their way around in communities and to approach people with presumed TB
**Patient**	1	Appreciation of ACF	x		Appreciation of ACF (e.g. for the benefits listed under “Innovation” above) motivated participation in ACF
	2	Willingness to participate in ACF	x	x	Community health workers used their experience and skills (see “Experience and skills” above) to convince people to participate in ACF
	3	Stigma and discrimination		x	Community health workers took sputum samples privately, e.g. in people’s homes, maintained confidentiality of people with TB and educated people and communities about the disease
	4	Trust and mistrust	x	x	Community health workers applied experience and skills (see “Experience and skills” above) to gain trust
**Social context**	1	Alcohol misuse		x	Community health workers advised people against alcohol misuse
	2	Community support	x		Community health workers involved members of the community to identify people with presumed TB and/or to convince a person with presumed TB to participate in ACF; community health workers asked community leaders for support in identifying people with presumed TB; community members informed each other about the possibility to participate in ACF; family members provided direct support to people with TB
	3	Knowledge and awareness about TB	x		Community health workers spread knowledge and awareness about TB among persons with presumed TB and communities; suggestions were made to further increase knowledge and awareness about TB, e.g. in schools, via radio, television, or through gatherings and street drama
	4	Poverty		x	Suggestions were made to provide food and/or cash transfers to people with TB
**Organizational context**	1	Collaboration with other stakeholders	x	x	Community health workers helped laboratory personnel in preparing slides when needed; community health workers informed public health office, village elders and politicians about ACF; suggestions were made improve collaboration with public health offices and government services
	2	Distance, weather conditions and transportation		x	Community health workers gave each other confidence to overcome distances; IMPACT TB provided transportation to support community health workers where possible; suggestions were made to provide equipment such as umbrellas, raincoats, torches, shoes, boxes for sputum transportation, mobile phones as well as lunch
	3	High workload		x	District Program Coordinators provided support to community health workers; community health workers took over laboratory tasks when needed; community health workers who are engaged in different projects (such as Female Community Health Volunteers) integrated their tasks, e.g. when going to a community they did not only conduct ACF, but did check-ups for pregnant women; suggestions were made to hire additional staff, e.g. to provide patient support
	4	Incentives for community health workers	x	x	IMPACT TB provided monetary incentives for ACF including travel costs; suggestions were made to increase monetary incentives
	5	Team support	x	x	Mutual support existed between Community Mobilizers, volunteers and laboratory staff; District Program Coordinators provided support to community health workers, e.g. though motivational talks
	6	Training	x	x	IMPACT TB included training for community health workers; suggestions were made to provide more training on TB

### Innovation

Interviewees stressed the benefits for ACF including access to healthcare, early detection, economic benefits, free testing and treatment, and support for people with TB. We grouped these into five categories.

In terms of access to healthcare, early detection of TB and patient support, interviewees outlined how ACF helped find ‘hidden patients’ who may have otherwise not gained healthcare access at all or too late. ACF spared people from difficulties, e.g. people did not have to travel great distances in accessing healthcare but were provided with care at their doorstep. Moreover, people with TB received counselling and treatment support.

*It (ACF) is a happy thing for us. If she (the volunteer) hadn’t come to our home, till now – either because of my stubbornness or I would have stayed idealistic assuming nothing has happened to me – my condition would have worsened and I would have been unable to go to health post.* (interviewee #6, person with TB)

*Now you people come house-to-house and take this trouble, now, this is good.* (interviewee #4, person with TB)

The economic benefits of ACF were portrayed as important. For example, a few people expressed that without ACF people with TB would have kept ‘roaming’ around in search of a diagnosis, costing them time and money, while their health status would deteriorate.

*If (the volunteer) would not have come, then I would have kept roaming, would take medicines and tablets, would be cured or not. No one knew, but when (the volunteer) came he knew about my problems and did my check-up (ACF).* (interviewee #11, person with TB)

Getting free testing and treatment was said to be a major benefit of ACF: “*Medicine doesn’t cost money, so it is easier”* (interviewee #8, person with TB), and *“the patient can be cured and earn and eat”* (interviewee #14, person with TB). At the same time, getting free testing and treatment served as an argument to convince people to partake in ACF. This underlines the fact that although TB testing and treatment is provided free in the government health service, patients incur significant costs in accessing the tests and treatment, which are ameliorated by ACF.

### Individual professional

Interviewees stressed the importance of the dedication and motivation of community health workers, their experience and skills, and having a network that facilitates ACF. These three categories are further detailed in the following.

Interviewees often described the dedication and motivation of community health workers as a strong facilitator for the implementation of ACF, e.g. in terms of taking the time to identify people with presumed TB, gain their trust and convince them to participate in ACF.

*Other people can survive because we are walking and working for them.* (interviewee #7, Female Community Health Volunteer)

What motivated community health workers were the ability to help people, families, communities and their country, as well as the feeling of being known and respected in society. There were a few cases where motivation was thought to be lacking: “*There are some who are (Community) Mobilizers only in the name.”* (interviewee #1, volunteer).

Experience and skills were crucial for the successful implementation of ACF. On the one hand, experience was about knowing the context, society and community, which allowed community health workers to adapt and *“move as per the society”* (interviewee #1, volunteer). Experience also included former work experience, e.g. as social worker or teacher, which could help community health workers find their way around in a community and identify people with presumed TB due to the networks, reputation and relationships they had previously built.

*I was a teacher from the beginning (…) and in the community, I have made a good impression from the beginning because I mix with people in community very well.* (interviewee #15, volunteer)

*It is easy for me. I have been working as a social worker for almost 15-20 years.* (interviewee #17, Community Mobilizer)

On the other hand, skills included communication, persuasion and interpersonal skills. Community health workers explained the ACF process to people with presumed TB and encouraged them to participate. In *Chitwan* and *Dhanusha* districts, community health workers highlighted the use of Xpert® MTB/RIF to help convince people with presumed TB to participate in ACF, e.g. one volunteer said: *“Only lucky people get this opportunity (to get tested with Xpert)”* (interviewee #1). Interviewees affirmed that the way community health workers talked to people was key. Sometimes the community health workers would have lunch or tea with prospective ACF participants to *“motivate them, befriend them ( …*) *calm them, make them happy”* (interviewee #9, volunteer). Wearing a vest with “BNMT TB” written on it was another way of communicating their mission, which helped ACF implementation.

*We need to absolutely get along with them (people in the community). We need to win their hearts. We need to listen to their hearts. Then only you will get the fruit for the work you are doing for the community.* (interviewee #5, Female Community Health Volunteer)

### Patient

Interviewees emphasized the importance of appreciation of ACF; willingness to participate in ACF; prevalence of stigma, discrimination and fear; as well as trust and mistrust as facilitators and/or barriers for ACF implementation. We grouped those factors into four categories.

The appreciation of ACF served as a facilitator for ACF implementation. Appreciation was expressed by people with TB, in particular, who thanked the community health workers and asserted how grateful they were for learning about TB, for having been diagnosed, and for having been provided with healthcare and support.

*I couldn't find out about it (TB) at the beginning, so lots of money was spent. Not even a single doctor said anything about it (TB). (The volunteer) is really a God for a person like me.* (interviewee #12, person with TB)

Interviewees elaborated on people’s willingness to participate in ACF. In some instances, people with presumed TB were not willing to participate, saying they did not have symptoms. At other times, people with TB did not want to enrol in TB treatment or did not complete the treatment course. Community health workers took time to explain why ACF participation and enrolling on treatment and continuing on it, was important.

Stigma and discrimination were among the reasons given for unwillingness to participate in ACF or to remain on TB treatment; being associated with TB could lead community members to look down on them, talk about them badly or stay away from them.

*The community will look down on them (people with TB). The community will discriminate them. They (the community) will say: “You are infected. Oh, don’t come near our house!” (…) They (people with presumed TB) might not want to or cannot talk on the road (about ACF). Like, they might be with their friends to whom they don’t want to share. We go to their house and also collect (the sputum sample).* (interviewee #5, Female Community Health Volunteer)

Community health workers therefore stressed the importance of maintaining confidentiality and educating people about TB, especially among those social classes (castes) considered “lower” than others.

Trust and mistrust in community health workers were alluded to as facilitators and barriers for ACF respectively. Trust led people with presumed TB to participate in ACF. Trust also led community members to share concerns about others in the community who may have TB. Mistrust was often caused by misperceptions, e.g. that the sole intention behind taking sputum samples was for community health workers to earn money.

*Rather than asking about our aim, they (people in the community) ask us how much of a stipend we get: “You would not take the sputum without any favour. (…) How can you do such work? How much money do you get?” We tell them: “No, we don’t get anything. We are FCHVs (Female Community Health Volunteers), we get the travel fare. We get lunch. We get trainings, that’s it. We don’t get big sum of money. We are doing it because it is our duty of being FCHVs. Not any random person can do this.* (interviewee #7, Female Community Health Volunteer)

### Social context

The social context was said to facilitate or challenge ACF implementation. We identified four categories based on the data: alcohol misuse, poverty, community support, and knowledge and awareness about TB.

Alcohol misuse was characterized as a barrier for ACF implementation, including for completion of TB treatment. A community health worker said that it was difficult to convince a person who was drunk to participate in ACF or remain on TB treatment, and that she would advise people to quit drinking for the sake of their health. Some people with TB described a relation between their drinking habits and their socioeconomic situation.

Due to poverty, people affirmed they could not afford food, or any expenses related to healthcare for themselves or their families. Suggestions were made to provide further support; a person with TB stressed: *“I want to eat nutritious foods ( …) but I don’t have money to buy and eat.”* (interviewee #13). A volunteer from the same district confirmed the need for *“financial support and support for having nutritious food ( …) for travel”* (interviewee #16).

*We are poor. This disease attacks to the poor only (…). We do smoking, take alcohol, isn’t it? We can’t afford food and medicines, can’t afford for the fee of doctors and hospitals. I have only one suggestion and that is, if we get help from anyone, anywhere then that would be good. Whatever we get as help, that would be good. (…) I get help for my treatment. I get medicines to save myself from such a dangerous disease. I shouldn’t go anywhere for the check-up; I get it at home. (…) Do I tell them (community health workers) to give me money? I don’t say it like that. That is not my nature. They are doing my treatment by coming to my home; it’s enough and great help for me.* (interviewee #12, person with TB)

On the one hand, community support was portrayed as support between community members. For instance, a person with TB heard others coughing and thus informed them about ACF. Family support for people with TB was also reported as important, e.g. in terms of being supportive, providing food and ensuring medicines were taken in time.

*I recently told two people. They were coughing, so I asked them to examine (for TB), saying nothing will go wrong: “If you are infected then you can take medicines and get cured, if not then it is not a problem. They test only sputum, not blood. (…) It doesn’t cost you money as well. Now you don’t need to waste whole day to take it (medicines) also, they (community health workers) will come to your doorsteps”. So, two people sent (sputum). Now that is because a person gets influenced by the environment around town. That perhaps makes the difference.* (interviewee #2, person with TB)

On the other hand, community support was also outlined in terms of support for community health workers in doing ACF. For example, community members contacted a community health worker directly to tell him/her about a person with presumed TB. In a community of a social class (caste) considered “lower” than others, a community health worker approached the community leader to gain support in identifying people with presumed TB, i.e. the community leader would be able to identify people and explain to them the reasons to participate in ACF.

Interviewees elaborated on the importance of knowledge and awareness about TB for ACF implementation, and, at the same time, the significance of ACF implementation to increase knowledge and awareness. A community health worker stated that *“the most important thing is that there is a lack of awareness (about TB) in the society”* (interviewee #15, volunteer). People may not know about the disease itself, about the availability of free treatment and the possibility of getting cured; interviewees pointed out the generally low education levels in some communities. People who did not know about TB were said to be reluctant to participate in ACF. Community health workers played a major role in spreading knowledge and raising awareness about TB, and people with TB also educated others about their disease.

*I think a programmes should be conducted to raise awareness and to inform the people at first around the village. (Street) drama can be shown. If we inform them then it will be easy to work in the community.* (interviewee #10, Female Community Health Volunteer)

### Organizational context

Interviewees emphasized the importance of collaboration with other stakeholders; distance to health centers, weather conditions and transportation; high workload; incentives for community health workers; team support; and training for ACF implementation. These six categories are further illustrated in the following.

Collaboration with other stakeholders was explained in terms of collaboration with laboratory staff who complained about the additional workload and, maybe as a consequence of that, were suspected by some community health workers of discarding sputum samples, or were thought to report test results late or not at all. Interviewees also talked about collaboration with staff at health facilities who they perceived as having negative attitudes and were sometimes absent, impacting treatment monitoring among people with TB. Government health services in Nepal are frequently crippled by lack of funding and human resources and infrastructure issues, political disruption and competing demands can lead to frequent service interruption. Interviewees stated that they needed more support from public health offices and better coordination with government services.

*They (staff at health facility) should do (provide TB treatment). And when we go there, we do to our level best. And when we go there, they (staff at health facility) tell us that our patients have not taken medicine for 3, 4, 5 days and ask us to go and talk to them. So, we feel sad. (…) We cannot talk to them too much, as we need to work together in the community.* (interviewee #17, Community Mobilizer)

Interviewees commented on how distance, weather conditions and transportation influenced ACF implementation. Even in the districts that are considered lowland plains, community health workers had to climb hills and overcome great distances and difficult terrain, walking for hours to get to their destination. Transportation services were seldom available. One volunteer expressed his appreciation about having been provided with a motorcycle to travel, while a Female Community Health Worker noted that she was not able to drive and would not be able to benefit from a vehicle. A Community Mobilizer stated that male community health workers were sent to areas further away compared to female community health workers. Both rainy and dry seasons brought difficulties, in terms of rising rivers that made routes impassable, as well as sun and heat that led to exhaustion. One interviewee suggested that the provision of mobile phones could facilitate ACF implementation.

*If there is mobile, we can get their number from home and call them. We can call them and ask them about the condition of patient in their village. We can even ask if the certain kind of diseases are present in their village or not. We can take number of one person from that far away community. When we have the phone number, we can request brother or sister from there to ask questions about the diseases till I reach there.* (interviewee #7, Female Community Health Volunteer)

Interviewees elaborated on equipment that could facilitate ACF implementation, such as umbrellas, raincoats, torches, shoes, boxes for sputum transportation, masks and gloves in greater quantities, new bags (as some of those provided had been torn over time), as well as lunch. Some also underlined the need for better transportation, e.g. through vehicles.

*We don’t even have an umbrella to protect us from sun and rain. We return only at night, but we don’t even have a light in our hand. (…) We wear slippers and go there, but it gets stuck in slippery mud and gets torn out. So, if you give us slippers, umbrella, shoes for our convenience, it would be of great help to us – for us, people who walk.* (interviewee #7, Female Community Health Volunteer)

Community Volunteers, Female Community Health Volunteers and Community Mobilizers alike described having a high workload, e.g. in terms of not having free time anymore or not having enough time to eat. The lack of transportation mentioned above also increased the workload, as community health workers needed to travel great distances. Moreover, the high workload meant that community health workers had to help out with additional tasks related to ACF, e.g. preparing samples for the laboratory when the laboratory was unstaffed. Female Community Health Volunteers were typically involved in a variety of health projects leading to additional workload. One Female Community Health Volunteer revealed how, while doing ACF, she would also use the opportunity to counsel pregnant women. Some interviewees felt that more staff was needed, e.g. laboratory workers and volunteers, especially to provide additional patient support. *“When they (community health workers) cannot work hard, when they cannot handle (ACF), then they leave”* (interviewee #3, Community Mobilizer). At the same time, one interviewee thought there were too many Community Mobilizers, leading to inefficiencies at work.

The importance of monetary incentives for their work was also stressed by Community Volunteers, Female Community Health Volunteers as well as Community Mobilizers. While appreciation for the existing incentives was expressed, interviewees also sometimes viewed these as insufficient or unfair. According to the interviewees, incentives should be increased, especially for long-distance travel, or that a salary be given.

*What (incentive) is for a lab person, and for volunteers is different. They (volunteers) are hurt because of this, a lot. They say that they do the hard work and lab people test in one place, and the incentive is not as per the work.* (interviewee #3, Community Mobilizer)

Team support was expressed as important for ACF implementation, e.g. to give each other confidence when travelling long distances, to guide each other in communities unknown to some, or to compensate for the high workload. Support from the District Program Coordinators was appreciated, e.g. in terms of showing respect and motivating community health workers in their work.

*Support comes from all sides. There is support from the lab. We have support from volunteers also. Now volunteers are working in the field; they are doing hard work. We have supported them, and they have also supported us.* (interviewee #3, Community Mobilizer)

Training for ACF implementation was highly valued. Interviewees appreciated the opportunity to learn about TB. In addition, one community health worker mentioned how, whenever he had a question related to ACF, he would ask the District Program Coordinators, read in books, or check on the internet. Interviewees articulated the need for additional training to expand and deepen their knowledge about TB.

## Discussion

This qualitative study investigated the perceptions of community health workers and people with TB regarding the facilitators and barriers for ACF implementation in four districts of Nepal. We generated five main themes from the data: (1) ACF addressed social determinants of TB by providing timely access to free healthcare, (2) knowledge and awareness about TB among people with TB, communities and community health workers were the ‘oil’ in the ACF ‘machine’, (3) trust in community health workers was fundamental for implementing ACF, (4) community engagement and support had a powerful influence on ACF implementation and (5) improved working conditions and better collaboration with key stakeholders could further facilitate ACF. These themes covered a variety of facilitators and barriers, which we divided into 22 categories that span across five framework levels (i.e innovation, individual professional, patient, social context and organizational context).

Our results are in line with available literature in the field, which has also pinpointed the importance of factors such as staff experience, motivation, workload and collaboration, as well as stigma and discrimination, mistrust, knowledge and awareness for ACF implementation [20–22, 30, 31]. In Nepal, mistrust of people with TB and their families has previously been reported as a key barrier for ACF implementation [32] and for accessing TB treatment [33]. These factors have been summarized in a recent scoping review on factors influencing ACF policy development and implementation [19]. Knowledge of both facilitators and barriers, and how to address them is essential for planning and implementing effective ACF. Involving multiple stakeholders in developing strategies on how to utilize facilitators (e.g. providing economic benefits) and overcome barriers (e.g. addressing stigma to increase participation in ACF) could furthermore optimize ACF implementation and scale-up. Finally, the similarity of our results compared to available evidence suggests possibilities for cross-learning, not only with regards to what the facilitators and barriers for ACF are, but in terms of strategies on how-to address these factors.

A direct comparison of this study with our findings in Vietnam allowed us to see similarities but also subtle differences between the two country contexts [Biermann O, Tran PB, Forse RJ, Vo LNQ, Codlin AJ, Viney K, Caws M, Lönnroth K. Capitalizing on facilitators and addressing barriers when implementing active tuberculosis case-finding in six districts of Ho Chi Minh City, Vietnam: a qualitative study with key stakeholders. Submitted. Despite the contextual differences between Nepal and Vietnam, many similar facilitators and barriers for ACF implementation were identified, e.g. community health workers’ dedication and motivation, experience and skills and having a network. Community health workers in Nepal and Vietnam capitalized on those strengths to address barriers at the “patient level” that were the same in both countries: stigma, discrimination and mistrust. Thus, in both Nepal and Vietnam, community health workers have a key role in ACF implementation and for it to be more effective and sustainable, they must therefore be placed at its center, alongside people with TB. While the perceived benefits of ACF were similar in both countries (e.g. access to healthcare, free testing and treatment and patient support), interviewees in Nepal also mentioned the economic benefit of ACF. Factors at the “social context level” were context-specific and included poverty and community support in Nepal. Moreover, alcohol misuse was mentioned as a barrier for ACF in Nepal, as it was difficult to convince a person who was drunk to participate in ACF or remain on TB treatment. In Vietnam, factors at the” "social context level” were engagement, commitment and support among stakeholders. At the “organizational context level”, factors such as distance, weather conditions and transportation were important in Nepal, less so in Vietnam where ACF was conducted in an urban area only. In both Nepal and Vietnam, interviewees appreciated and requested increased incentives and training.

This study not only shed light on practical facilitators and barriers for ACF implementation, but also brought to light the importance of the social determinants of TB, including structural and intermediary determinants [34]. Important structural determinants that seemed to have influenced ACF implementation according to our data included social class (caste), gender, education and income. Meanwhile, intermediary determinants such as living and working conditions, behaviors and psychosocial factors also seemed to have impacted ACF implementation. Many of those determinants not only mattered for people with TB, but for community health workers. For example, gender mattered in the case of a Female Community Health Volunteer who was not able to drive a motorcycle, while a male volunteer was able to and rode a motorcycle to distant areas. Moreover, social class and power dynamics played a role, e.g. when community health workers collaborated with staff from health facilities or when they approached people with presumed TB in communities. Importantly, power dynamics should never be exploited to force people with presumed TB into participating in ACF. ACF implementation cannot be fully successful as long as the underlying barriers related to the social determinants of TB prevail. The social determinants of TB influence ACF implementation, while ACF can also influence the social determinants in return. Action is needed to address all structural and intermediary determinants of TB alike, for example by providing people with TB with socioeconomic and psychological support [35, 36]. Limited socioeconomic support for MDR-TB affected households in Nepal is provided by the national TB Programme and multiple NTP partners are involved in efforts to strengthen these support programmes in the future [37].

### Strengths and limitations

A strength of this study is the inclusion of community health workers as well as people with TB, who provided insights into the IMPACT TB ACF implementation. Moreover, KD and BR were experienced data collectors who were familiar with the Nepali context, including ACF and the Nepali health system, which helped generate high-quality, contextualized data for our study. In addition, the triangulation of data and extensive discussion among members of the research team and the BNMT team enabled us to better understand and ascertain the dependability, accuracy, breadth and depth of the data collected. This study adds value to existing knowledge by providing concrete insights into how to strengthen facilitators and how to overcome barriers related to ACF implementation. While MC and KL had been involved in the planning of IMPACT TB, KD and BR had been closely involved in planning and/or implementation. OB and KV did not have any role in planning or implementing IMPACT TB. The inclusion of both outsider and insider perspectives on data collection and analysis strengthened the study, while the insider perspective of some members of the research team may have also introduced bias.

Some limitations of this study are selection and social desirability bias. KD and BR tried to mitigate social desirability bias by building rapport with the interviewees and by following up statements made by interviewees with clarifying and probing questions during the interviews rather than strictly adhering to the interview guides. Moreover, we did not conduct interviews with District Program Officers, laboratory personnel, health workers in TB treatment facilities or leaders of District TB and Leprosy Units. Their perspectives could have added important information to this study; we hope that future research will fill this gap. Finally, interviewees’ rationale for implementing/participating in ACF was not always clear, which may affect the reliability of the data.

### Future research

This study identified a wide range of facilitators and barriers as well as “how-to” strategies for ACF implementation. At the same time, this study also uncovered topic areas for future research to address, e.g. to further contextualize, integrate and maximize the impact of ACF interventions, a more in-depth understanding of the social determinants of TB and the “social context level” (including factors such as alcohol misuse, poverty, knowledge and awareness about TB, and community support) is needed. The perspectives of people, especially those who were unwilling or unable to participate in ACF, should be further researched, e.g. focusing on how trust in community health workers and health care is built and maintained over time. Lessons about trust may also be learned through evidence syntheses from other infectious diseases, including Ebola outbreaks and the COVID-19 pandemic, which have highlighted the crucial importance of public trust for successful public health interventions. Anthropological studies and observations may further be able to fill this gap. Finally, we need research and action to improve the working conditions and integration of community health workers into the health system.

## Conclusion

This study provides new insights into facilitators and barriers for the implementation of ACF within the IMPACT TB project in Nepal, as perceived by community health workers and people with TB. The results emphasize the importance of addressing the social determinants of TB, while key ingredients for ACF implementation include knowledge and awareness of TB, trust in community health workers, community engagement and support, as well as good working conditions and stakeholder collaboration. All of these ingredients are required for successful ACF implementation, while the absence of these factors may convert them from facilitators into barriers for ACF. The similarity of our results compared to results from IMPACT TB in Vietnam and other available evidence suggests possibilities for cross-learning, not only with regards to what the facilitators and barriers for ACF are, but in terms of strategies on how to address these factors. The findings can thus inform the planning and implementation ACF in Nepal and similar contexts in low- and middle-income countries worldwide.

## Supplementary Information

**Additional file 1.**

**Additional file 2.**

## Data Availability

The datasets generated and analysed during the current study are not publicly available. The informed consent that all interviewees signed promised full anonymity. Following data requests, survey transcripts will be reviewed for any potential identifying information and will only be made available to researchers who sign a data sharing agreement. Data requests may be sent to the corresponding author.

## Abbreviations

ACF: Active case-finding
BNMT: Birat Nepal Medical Trust
COREQ: COnsolidated criteria for REporting Qualitative research
HIV: Human Immunodeficiency Virus
IMPACT TB: Implementing proven community-based active TB case-finding intervention
TB: Tuberculosis
WHO: World Health Organization
