# Supplementary material for: Building on facilitators and overcoming barriers to implement active tuberculosis case-finding in Nepal, experiences of community health workers and people with tuberculosis
Source: BMC Health Serv Res. 2021 Apr 1;21:295. doi: 10.1186/s12913-021-06290-x (PMC8015737; doi:10.1186/s12913-021-06290-x)
Supplement: Supplementary file 2 — Additional file 2. [file 12913_2021_6290_MOESM2_ESM.docx]

Building on facilitators and overcoming barriers to implement active tuberculosis case-finding in Nepal, experiences of community health workers and people with tuberculosis

Olivia Biermann¹*, Kritika Dixit¹ ², Bhola Rai², Maxine Caws² ³, Knut Lönnroth¹, Kerri Viney^4^

^1^ Department of Global Public Health, WHO Collaborating Centre on Tuberculosis and Social Medicine, Karolinska Institutet, Tomtebodavägen 18a, 17177 Stockholm, Sweden

^2^ Birat Nepal Medical Trust, Lazimpat, Kathmandu, Nepal

^3^ Department of Clinical Sciences, Liverpool School of Tropical Medicine, Pembroke Pl, Liverpool L3 5QA, United Kingdom

^4^ Research School of Population Health, College of Health and Medicine, Australian National University, Canberra, Australia

*Corresponding author
Email: olivia.biermann@ki.se

**Additional file 2 – Interview guide for key-informant interviews (with community health workers and people with TB)**

*The objective of this study is to describe main facilitators and barriers for IMPACT TB’s ACF implementation and implementation strategies.*

1. What should be done for early diagnosis of TB?
2. Could you please describe your experience in implementing ACF/when the volunteer came to your house to test you for TB?
3. From your perspective, what are the benefits of ACF?
4. And what would you consider risks of ACF?
5. Which would you consider the single most important facilitator in implementing/participating in ACF?
   - Thinking about the overall health system, including resources, incentives, management structures, turnover/access, etc.
   - Thinking about the/your community, including your neighbors, etc.
   - Thinking about yourself and any barriers in putting ACF into practice/participating in ACF yourself
   - Were there any other major facilitators?
6. Were you able to strengthen/take advantage of facilitator x *(note: Ask about the facilitator that the interviewee considered most important.)*?
   - If yes, how?
   - If no, why not?
7. What should be done to strengthen/take advantage of facilitator x in the future *(note: Ask about the facilitator that the interviewee considered most important.)*?
   - **How** can this strategy be put in practice?
8. Which would you consider the single most important barrier in implementing/participating in ACF?
   - Thinking about the overall health system, including resources, incentives, management structures, turnover, etc.
   - Thinking about the/your community, including your neighbors, etc.
   - Thinking about yourself and any barriers in putting ACF into practice/participating in ACF yourself
   - Were there any other major barriers?
9. Were you able to overcome barrier x *(note: Ask about the barrier that the interviewee considered most important.)*?
   - If yes, how?
   - If no, why not?
10. What should be done to overcome barrier x in the future *(note: Ask about the facilitator that the interviewee considered most important.)*?
    - **How** can this strategy be put in practice?
11. What would be an important action that *others* (e.g. healthworkers, colleagues) could do better to implement ACF?/ What would be an important action that *others* (e.g. your neighbors, friends and family) could do to support you in participating in ACF?
12. What would be an important action that *you* personally can do better to implement/participate in ACF?
13. (For FCHVs: How do you prioritize ACF amongst other tasks you have?)
14. Do you have any additional comments?
